# Genetic pleiotropy underpinning adiposity and inflammation in self-identified Hispanic/Latino populations

**DOI:** 10.1186/s12920-022-01352-3

**Published:** 2022-09-10

**Authors:** Mohammad Yaser Anwar, Antoine R. Baldassari, Hannah G. Polikowsky, Colleen M. Sitlani, Heather M. Highland, Nathalie Chami, Hung-Hsin Chen, Mariaelisa Graff, Annie Green Howard, Su Yon Jung, Lauren E. Petty, Zhe Wang, Wanying Zhu, Steven Buyske, Iona Cheng, Robert Kaplan, Charles Kooperberg, Ruth J. F. Loos, Ulrike Peters, Joseph B. McCormick, Susan P. Fisher-Hoch, Christy L. Avery, Kira C. Taylor, Jennifer E. Below, Kari E. North

**Affiliations:** 1 Department of Epidemiology, University of North Carolina at Chapel Hill, 123 West Franklin Street, CVD Genetic Epidemiology Lab, Fl #4, Room A7, Chapel Hill, NC 27599 USA; 2 Vanderbilt Genetics Institute, Vanderbilt University Medical Center, Nashville, TN 37232 USA; 3 Department of Medicine, University of Washington, Seattle, WA 98195 USA; 4 The Charles Bronfman Institute for Personalized Medicine, Icahn School of Medicine at Mount Sinai, New York, NY USA; 5 Department of Biostatistics, University of North Carolina at Chapel Hill, Chapel Hill, NC 27599 USA; 6 Carolina Population Center, University of North Carolina at Chapel Hill, Chapel Hill, NC 27516 USA; 7 Translational Sciences Section, School of Nursing, University of California, Los Angeles, Los Angeles, CA 90095 USA; 8 Jonsson Comprehensive Cancer Center, University of California, Los Angeles, Los Angeles, CA 90095 USA; 9 Department of Statistics, Rutgers University, Piscataway, NJ 08854 USA; 10 Department of Epidemiology and Biostatistics, Helen Diller Family Comprehensive Cancer Center, University of California San Francisco, San Francisco, CA 94115 USA; 11 Albert Einstein College of Medicine, Bronx, NY 10461 USA; 12 Division of Public Health Sciences, Fred Hutchinson Cancer Center, Seattle, WA 98109 USA; 13 School of Public Health, University of Texas Health Science Center at Houston, Brownsville Regional Campus, Brownsville, TX 78520 USA; 14 Department of Epidemiology and Population Health, University of Louisville School of Public Health and Information Sciences, Louisville, KT 40202 USA

**Keywords:** Obesity, Inflammation, Genetic pleiotropy, Hispanic Americans

## Abstract

**Background:**

Concurrent variation in adiposity and inflammation suggests potential shared functional pathways and pleiotropic disease underpinning. Yet, exploration of pleiotropy in the context of adiposity-inflammation has been scarce, and none has included self-identified Hispanic/Latino populations. Given the high level of ancestral diversity in Hispanic American population, genetic studies may reveal variants that are infrequent/monomorphic in more homogeneous populations.

**Methods:**

Using multi-trait Adaptive Sum of Powered Score (*aSPU*) method, we examined individual and shared genetic effects underlying inflammatory (CRP) and adiposity-related traits (Body Mass Index [BMI]), and central adiposity (Waist to Hip Ratio [WHR]) in HLA participating in the Population Architecture Using Genomics and Epidemiology (PAGE) cohort (N = 35,871) with replication of effects in the Cameron County Hispanic Cohort (CCHC) which consists of Mexican American individuals.

**Results:**

Of the > 16 million SNPs tested, variants representing 7 independent loci were found to illustrate significant association with multiple traits. Two out of 7 variants were replicated at statistically significant level in multi-trait analyses in CCHC. The lead variant on *APOE* (rs439401) and rs11208712 were found to harbor multi-trait associations with adiposity and inflammation.

**Conclusions:**

Results from this study demonstrate the importance of considering pleiotropy for improving our understanding of the etiology of the various metabolic pathways that regulate cardiovascular disease development.

**Supplementary Information:**

The online version contains supplementary material available at 10.1186/s12920-022-01352-3.

## Introduction

Self-identified Hispanic/Latino Americans (hereafter Hispanics) harbor an elevated burden of obesity (> 40%) [1]. Excess body weight is a known risk factor for a range of metabolic abnormalities, including insulin resistance [2], high blood pressure [3], dyslipidemia [4], and metabolic hormones disorders [5].

The associations between obesity and metabolic irregularities are often attributed to induction of inflammatory responses by adipose tissue [6, 7]. Fat accumulation leads to enlargement (hypertrophy) of adipocytes with impaired capacity for fat oxidation, glucose regulation and amelioration of inflammatory activities [8, 9]. Hypertrophied adipocytes exhibit a distorted cytokine secretion profile [7, 10]; cytokines are key drivers of C-reactive protein (CRP) production [11], which is associated with cardiovascular diseases (CVD) [12], and a significant predictor of CVDs’ clinical course [13]. Clinical studies suggest lowering serum CRP levels significantly reduce CVD incidences [14].

Although this process has been highlighted as the presumptive pathophysiological direction of adiposity-CVD risk factors association, compelling evidence also suggests that inflammation could induce adiposity. For example, inflammation-induced insulin resistance may trigger hyperplastic and hypertrophic changes in adipose tissues to alleviate glucose accumulation [15]. Furthermore, results from a longitudinal study illustrated steeper weight gain among those with higher baseline CRP levels in a 3-year follow-up period [16]. More corroborating evidence for adiposity-inflammation interrelatedness has emerged with the increased availability of genetic studies. High levels of inflammatory markers in mice carrying genes that increase obesity [17], or concurrent inflammatory effects where the expression levels of adipose tissue genes were regulated [18, 19], all support common molecular pathways for inflammation and obesity.

These common pathways [20] suggest pleiotropic disease underpinnings [21], which may inform classification and treatment of disease. Yet, exploration of pleiotropic variants in the context of adiposity-inflammation has been scarce [22], and none have included Hispanics populations. Given the high level of ancestral diversity in Hispanics, genetic studies may reveal variants that are infrequent/monomorphic in more homogeneous populations [23, 24]. Furthermore, the differential distribution of inflammatory markers associated with comparative adiposity configurations [25, 26], patterns of fat accumulation [27, 28], and the prevalence of cardiometabolic abnormalities in Hispanics [29] highlights the need for adiposity-inflammation pleiotropy study in this underserved group.

We, therefore, examined individual and shared genetic effects underlying inflammatory (CRP) and adiposity-related traits (overall obesity: Body Mass Index [BMI], and central adiposity: Waist to Hip Ratio [WHR]) in self-identified Hispanics participating in the Population Architecture Using Genomics and Epidemiology (PAGE) study with replication of effects in the Cameron County Hispanic Cohort. For multi-trait analysis, we used adaptive Sum of Powered Score (*aSPU*) [30] method. Advantages of *aSPU* over similar methods include its ability to accommodate differences in sample size between traits and phenotypic distributions, preserved type 1 error in select scenarios, where some other methods did not [30], performance in the context of heterogeneity in direction of phenotypic effect, and scalability and computational efficiency which enabled examination of millions of variants in a reasonable time.

We also assessed whether replicated signals were directly associated with relevant phenotypes (i.e., biologic pleiotropy) or indirectly associated as mediators (i.e., mediated pleiotropy). Assuming that adiposity and inflammation are interrelated manifestations of biological mechanisms, pleiotropy assessment may provide insights on the development of effective preventive strategies designed to improve the cardiovascular health of Hispanics. Our results illustrate the broad utility of pleiotropy assessment to illuminate the biology of complex diseases and traits.

## Materials and methods

### Study populations

The conceptual framework for the adiposity-inflammation pleiotropy study is displayed in Additional file 2: Fig. S1. We chose a discovery and replication study design of self-identified Hispanics study participants from the two studies. The PAGE study population includes several studies at different sites [31, 32] as described previously [33]. In brief, PAGE consists of multiple populations grouped by self-identified ethnicities: European Americans (Non-Hispanic whites [NHW]), African Americans (AA), Hispanics, American Indians (AI), East Asians (ASN), and Native Hawaiians/Pacific Islander (HAW). All participating sites in PAGE ascertained both men and women except for the women only Women’s Health Initiative (WHI). We studied the Hispanics sub-cohort as our discovery set (N = 35,871) from four studies within the PAGE: Hispanic Community Health Study/ Study of Latinos (HCHS/SOL), the Women’s Health Initiative (WHI), BioMe Biobank (BioMe), and MultiEthnic Cohort (MEC). Principal component analyses suggest substantial ancestral variability among self-identified Hispanics/Latinos in PAGE (Additional file 1: Appendix, Section 1. Fig. 1.). All study participants provided written informed consent and each study was approved by relevant institutional review board. The replication study included self-identified Hispanics study participants recruited at the US-Mexico border, the Cameron County Hispanic Cohort (CCHC). This cohort was established in 2004 and now numbers 5,000 individuals randomly selected from a population with severe health disparities. Many of the participants are originally from Mexico, and we have collected detailed information on participant place of birth, how recently they arrived in the US, income, marital status, and employment [34]. Out of 5000 individuals recruited for the cohort, a sub sample of 3,313 genotyped individuals are included in this study. The CCHC is almost exclusively self-report Mexican American, with substantial patterns of admixture from Native American and European ancestry, with limited African admixture (Additional file 1: Appendix, Section 1. Fig. 2.)

### Phenotype measurements and quality control

We studied two anthropometric phenotypes: BMI (kg/m^2^; a measure of overall adiposity) and WHR (proxy measure of central obesity). For all studies, except in MEC, height and weight were measured by study staff at study enrollment, to calculate BMI (weight/height^2^). In MEC, BMI is based on self-reported height and weight at enrollment. Pilot analyses of BMI in MEC illustrated a comparable distribution to national surveys [35]. Waist circumference was measured at the level of natural waist in horizontal plane to the nearest 0.5 cm [36]; no waist or hip measurement was available for BioMe sub-cohort; WHR measures were multiplied to 100 for ease of interpretation. Analyses of WHR measures were stratified by sex to account for well-established sexual dimorphism. For inflammation, we used high sensitivity CRP (hsCRP) as robust marker of systemic inflammation with strong association with central obesity [37]; CRP was measured at enrollment separately in each contributing cohort and subsequently harmonized for the entire set [38].

### Genotype and quality control

Most individuals (~ 20,000) were genotyped using the MEGA array panel; details on the genotyping were published previously [39]. The remaining PAGE samples were genotyped with Affymetrix and Illumina arrays. We used the genome-wide variants’ set that was imputed to 1000 Genome Phase 3 reference population after quality control (QC), and details are accessible [40]. For this study analyses, we next excluded genetic variants with poor imputation (R^2^ < 0.4), effective sample size of < 30, and minor allele frequency (MAF) of < 0.05; criteria used for calculating effective sample size for each single nucleotide polymorphisms (SNP) is defined in Additional file 1: Appendix, Section 2. Out of > 60 million SNPs, approximately 32 million SNPs were removed because of low MAF. The replication cohort from the CCHC was genotyped at the Vanderbilt University Medical Center genotyping core facility, VANTAGE, using MEGA-EX Array panel. After standard QC, imputation was completed using the Trans-Omics for Precision Medicine (TOPMed) [41] freeze 8 panel available on the NHLBI Imputation Server (https://imputation.biodatacatalyst.nhlbi.nih.gov), by having American ancestry as reference group. We used same QC criteria to exclude SNPs in this replication cohort as substudies from PAGE.

### Statistical analyses

#### Association tests

The skewed distribution of CRP necessitated log-transformation before regression analyses. Extreme observations (defined as values outside three standard deviations (SD) from the mean in the log transformed distributions (CRP), or 4SD for BMI and WHR) were flagged as outliers and excluded from all analyses. WHR for women and men were treated as separate phenotypes, so that the phenotypes included were CRP, BMI, WHR-men, and WHR-women. Residuals for each phenotype were estimated from linear regression models adjusted for age, age2, sex (when applicable), BMI (when applicable), center, cohort, and age-by-sex interaction, performed separately in each study. Residuals were then inverse rank normalized and used as the outcome for univariate genome-wide association tests (GWAS). All MEGA-genotyped samples were pooled together for genetic association testing while the remaining non-MEGA data were analyzed by study. GWAS were performed using SUGEN, adjusted for 10 genetic principal components (PC). SUGEN employs generalized estimating equations (GEE) to adjust for family relationships (first or second degree), with independent error distributions by self-identified group [42]. GWAS results from MEGA samples and other studies were then meta-analyzed with METAL [43] assuming fixed-effect inverse-variance weighting.

#### Multi-trait association test

Various multi-trait methods have recently been proposed [44]. Most of these methods offer superior statistical power compared with multivariate analysis [45]. To identify loci with evidence for associations with one or more of the adiposity and inflammation traits, we combined Z-score statistics from meta-analysis results from each phenotype using *aSPU* [30] test.

Briefly, *aSPU* aggregates information across *n* phenotypes for a given SNP by taking the sum of its univariate GWAS Z-scores *S*_*p*_, each raised to some power *γ*, so that a higher *γ* increases the influence of strongly-associated phenotypes on the score $SPU\left(\gamma \right)={\sum_{n}{(S}_{p})}^{\gamma }$ [30]. With *γ* taking one of many competing values (1, 2, …, 8), *aSPU* selects a maximally-efficient scheme to detect combined phenotype effects on the entire group of phenotypes. Monte-Carlo methods applied to univariate GWAS Z-scores estimated by inverse-variance-weighted meta-analysis are then used to generate *aSPU* P-values, which are interpreted as evidence for an association of the SNP with at least one phenotype. SNP-specific *γ* scores also provide a mechanism to group loci from most to least likely to be pleiotropic.

A set containing trait-specific GWAS-inferred Z-scores (from all contributing phenotypes) was constructed. Multi-trait analysis was performed using the *JaSPU* (*github.com/kaskarn/JaSPU*) package, with the number of iterations set to 100 billion. This program relies on a Markov Chain Monte-Carlo (MCMC) [46] iterative process to estimate p-values. The p-values generated from this test were used to assess statistical significance of whether SNPs were associated with one or more phenotypes.

We considered a SNP as a candidate for pleiotropy analyses if a): exceeded significance threshold for multi-trait test at *PaSPU* < 1.25 × 10^–8^, a conservative threshold [47] which accounted for multiple testing on likely low frequency SNPs and the number of traits tested, and b) nominally significant for inflammation and at least one adiposity trait in univariate GWAS results. We subsequently identified significantly associated loci where: i) variants in the region met the criteria above, and (ii) were present in pooled MEGA results; latter criterion was used to ensure understudied SNPs existed in both PAGE and CCHC results, thereby usable for likewise comparison. Linkage disequilibrium (LD) analyses identified independent variants (*R2* < 0.1). For each independent locus, we highlighted the most likely functional or closest observed proxy to functional variant based on a literature review, and the lowest or close to the lowest *PaSPU*.

#### Bioinformatic annotation

Ensemble Variant Effect Predictor (VEP) (*grch37.ensembl.org/*) was used to determine the effect of each variant on genes, transcripts, protein sequences, and regulatory regions. We searched replicated variants and their close LD proxies (with LD cut-point of R2 > 0.8) in the publicly available database *PhenoScanner* [48] and GWAS Catalogue [49] for reported associations with any phenotype including adiposity and inflammation traits at *P* < 1 × 10^–5^ significance level. LD clumping was performed using the AMR ancestry panel. We used both Gene Expression Portal (*GTEx* Portal) [50] and *PhenoScanner* to examine variants and close proxies (*R2* > 0.5) for functional significance and influence on tissue expression at GWAS significance level, which are genomic regions associated with expression levels of messenger RNA (mRNA). Finally, *Haploreg* was used to evaluate the regulatory potentials of variants on haplotype blocks, such as candidate regulatory SNPs at disease-associated loci [51]. This annotation tool was used to assess the effects of SNPs on regulatory motifs and expression Quantitative Trait Loci (eQTL).

#### Replication of shared genetic loci

All significant variants were taken forward to replication in the CCHC. GWASs were performed with SUGEN. Multi-trait analysis was completed following the same *aSPU* based approach used in the discovery stage. The criteria set for replication included, a) variant is nominally significant in multi-trait test (*Paspu* < 0.05), b) variant was nominally significant for CRP and at least one anthropometric trait, and c) direction of effect of each SNP was same in discovery vs replication cohort for the tested trait.

#### Causal pathway analyses

We performed a causal mediation pathway analyses of replicated variants to assess pleiotropic potential of identified variants, and whether an observed association is independently associated with several phenotypes and the genetic effect is transmitted through a common pathway that is upstream to the associated phenotypes (i.e., biologic pleiotropy, Additional file 2: Fig. S1); or induced due to relationships between the outcome phenotype and a “mediating” phenotype [53]. Elucidating these complexities could provide novel biological insights [54].

Mediation analyses allows for the assessment of whether a SNP has a direct effect on the phenotype of interest. The R package Mediation [55] was used for analysis, which conducts a three-step process. The total effect between a SNP and an outcome is assumed to be the sum of the average causal mediation effects (ACMEs) and the average causal direct effect (ADE). The first step includes estimating the distribution of the mediator phenotype as a function of the SNP, adjusted for genetic ancestry (e.g., mediator ~ genotype (variant) + PCs + covariates). The next step involves estimating the distribution of the outcome phenotype as a function of the mediator phenotype, genotype, and covariates (e.g., outcome ~ mediator + genotype + PCs + covariates). The final step combines the fitted models in the mediation equation, providing estimates and p-values for the direct (e.g., genotype → adiposity [outcome]) and indirect/mediating (e.g., genotype → mediator → adiposity [outcome]) associations. We did not allow for any mediator by genotype interaction [16]. We performed mediation analyses in the biggest subset of the discovery cohort that were genotyped by the same MEGA array, excluding samples with missingness since mediation method required complete observation across output and mediating phenotypes; the total available sample size stood at 15,600. We considered age and sex as covariates in the mediator and outcome models, together with first 10 PCs. We additionally performed sex stratified analyses but 95% confidence intervals and point estimates overlapped with sex adjusted results. With this method, quasi-Bayesian Monte Carlo simulations are used for the estimation of standard errors. The number of simulations was set to 1000, with robust standard errors using sandwich estimators [56]. We also used same mediation package to further perform sensitivity analysis on the mediated and direct effects for violation of the sequential ignorability assumption and assessing the effect of unmeasured confounders. For genotypes, we used allele dosages and models were estimated assuming additive genetic effects.

For each significantly replicated SNP, if a SNP was previously associated with inflammation, we performed mediation analyses assuming CRP as mediator and an adiposity trait as the outcome (Additional file 3: Fig. S2); conversely, where a SNP was primarily associated with adiposity in prior studies, then we used adiposity phenotype as the mediator and CRP as the outcome. Statistical significance of ADE in either scenario would suggest an independent association of the lead SNP with the outcome and a potentially biologic pleiotropy effect.

## Results

### Sample description

Most participants were women (N = 22,733 (63.4%) and the average age was 52.9 years (SD = 14; Table 1). Most study participants were overweight and centrally obese, with an average of BMI of 29.2 kg/m^2^ and WHR of 86.6 in the discovery group, and mean BMI of 30.8 kg/m^2^ and mean WHR of 91.2 in CCHC replication group. Inflammation was apparent with a mean value of CRP of 4.2 mg/dL in the discovery group. A small number of samples (N = 33 for BMI, N = 21 for WHR in females, N = 13 for CRP) were excluded as outliers.

**Table 1:** Descriptive Distribution of anthropometric and inflammation phenotypes in self-identified Hispanic/Latinos Americans sub-cohort of PAGE (discovery) and CCHC (replication) populations

Study	Sample size	Female (%)	Age (years, SD)	BMI (kg/m^2^, SD)	WHR in women (wc/hip × 100, SD)	WHR in men (wc/hip × 100, SD)	CRP (mg/dL, SD)
SOL	11,986	7026 (58.6)	46.1 (13.8)	29.8 (6.0)	90.0 (7.2)	95.4 (6.8)	3.9 (5.9)
BioME	10,727	6766 (62.2)	51.6 (16.2)	29.5 (6.6)	NA	NA	4.1 (4.4) ^a^
WHI	5386	5386 (100)	60.2 (6.8)	29.1 (6.3)	82.0 (8.0)	NA	5.0 (6.6)^b^
MEC	7772	3555 (45.7)	60.0 (7.1)	27.9 (4.8)	88.1 (7.6)^c^	95.6 (6.4)^d^	3.9 (4.4)^e^
Total Sample (Discovery study)	35,871	22,733 (63.4)	52.9 (14.0)	29.2 (6.0)	86.6 (8.4)	95.6 (6.8)	4.2 (6.8)
CCHC (Replication Study	3313	2074 (65.6)	45.9 (17.2)	30.8 (6.5)	91.2 (7.1)	95.5 (6.4)	6.2 (6.9)^f^

^a^(Missing 10,265), ^b^(Missing 1436), ^c^(Missing 1152), ^d^(Missing 1398), ^e^(Missing 3407), ^f^(Missing 1376)

### GWAS and multi-trait association analyses in PAGE

More than 16 million SNPs were available for analyses post QC and were evaluated in our combined phenotype analysis of 4 traits (BMI, WHR in men, WHR in women and CRP). No genomic inflation was observed in univariate GWAS (Additional file 5: Table S1). In univariate GWAS, we observed 3 independent loci (i.e., LD *R*^2^ < 0.1) exceeding genome-wide significance level (P < 5 × 10^−8^) for BMI, one locus for WHR in men and one for WHR in women, and 11 loci for CRP in meta-analyzed results (Additional file 5: Table S2). All observed loci were previously known. In subsequent multi-trait association assessment, SNPs representing 7 independent loci exceeded the multi-trait test threshold (*PaSPU* < 1.25 × 10^−8^) (Additional file 5: Table S3).

### Phenotype decomposition of multi-trait significant variants in PAGE

All SNPs except for rs11642015 (*FTO*) were associated with CRP at GWAS significance level; the *FTO* SNP was associated with BMI (Additional file 5: Table S3). SNPs rs11208712 and rs9987289 (both intergenic) were also associated with BMI at nominal level. SNPs rs62158854 (*IL1RN*), rs1169288 (*HNF1A*) and rs439401 (*APOE*) were nominally associated with WHR-women. The remaining rs12064564 on chromosome 1 was nominally associated with WHR-men.

### Functional assessments

*Phenoscanner* and GWAS-catalogue probe indicated that multi-trait significant SNP on chromosome 16 (rs11642015, overlapping *FTO* gene) and its close proxies (*R2* > 0.8) is primarily associated with anthropometric and fat mass traits (Additional file 4: Fig. S3, Additional file 5: Table S4). rs9987289 on chromosome 8 (*LOC157273*) is associated with lipid traits, liver enzymes, and glucose (diabetes) measures. rs1169288 on chromosome 12 (*HNF1A*), is primarily reported in association with lipid traits and CRP. rs11208712 (intergenic) on chromosome 1 is reported for blood immunity cells and CRP. Variant rs62158854 on chromosome 2 (IL1RN gene) is mostly associated with blood immunity cells. We found only one study reported rs12064564 on chromosome 1 (intergenic) associated with CRP. The APOE variant (rs439401, on chromosome 19) is frequently reported in association with lipid traits, and in some studies in connection with Alzheimer’s disease.

### Tissue gene expression

Probe of *GTEx*, and *PhenoScanner* for multi-trait significant variants suggested that except for rs12064564 and rs11208712 on chromosome 1, the remaining SNPs were associated with higher mRNA expression in several tissues including skin, thyroid, brain, adipose, artery, whole blood, esophagus, testis, and colon (Additional file 6: Fig. S4, Additional file 5: Table S5). SNP rs11208712 exhibited gene expression only with whole blood and pancreas, though not at GWAS significance level.

### Replication with CCHC

We carried forward the 7 loci for replication in the CCHC population. 2 of the 7 variants met replication criteria in CCHC multi-trait association results (Table 2, Additional file 5: Table S3): variant rs439401 (*APOE*) on chromosome 19, and rs11208712 (intergenic) on chromosome 1.

**Table 2:** Variants with evidence for genetic pleiotropy that were replicated in Cameron County Hispanic Cohort

Chromosome	Position (HG19)	PAGE multivariate *p* value	CCHC multivariate *p* value	MAF in Hispanics	MAF in Europeans	rsID	Gene
*1*	66148652	1.00E−11	0.007731	0.44	0.36	rs11208712	Intergenic
*19*	45414451	1.00E−11	0.0291	0.42	0.38	rs439401	APOE

### Regulatory significance of replicated multi-trait SNPs

We searched *Haploreg* for regulatory features for overlap of the replicated variant with enhancers with enrichment of histone 3 lysine 4 monomethylated 1 [H3K4me1] and H3K27 acetylation [H3K27ac], and promoter epigenomic markers with enrichment of H3K4me3 and H3K9ac. Lead SNP rs439401 (*APOE*) exhibited higher frequencies of enhancer and promoter annotations in the epithelial and brain tissues (Additional file 5: Table S6), enhancer annotations with embryonic stem cells, muscles, heart, digestive, and various cells with hormonal functionality. The remaining rs11208712 on chromosome did not illustrate regulatory annotation in Haploreg database.

### Causal mediation analyses

We performed mediation analyses for replicated SNPs. Primarily known for association with inflammation, we assumed CRP as mediator in association between rs11208712 and BMI (the phenotype at association with SNP at nominal level). Results suggest directly positive and indirectly negative (via CRP) associations of the SNP with BMI, though total effect (e.g., direct + indirect effect) was positive but marginally nonsignificant (beta = 0.12, 95% CI [− 0.01, 0.26]) (Table 3). In comparison, rs439401 (*APOE*) was previously associated with adiposity (WHR), and therefore was used as mediator in association between the SNP and CRP. Analyses suggested positive indirect (via WHR) and direct (independent of WHR) associations with CRP. These results were supportive of biologic pleiotropy effects for *APOE*. Results were estimated with robust standard errors and remained consistent after sensitivity tests.

**Table 3:** Results of mediation analyses

SNP	Gene	Mediator	Outcome	Direct effect (95% CI)	Direct effect *p* value	Mediated effect (95% CI)	Mediated effect *p* value	Total effect (95% CI)	Total effect *p* value
rs11208712	Intergenic	CRP	BMI	− 0.12 (− 0.24, 0.00)	0.044	0.25 (0.19, 0.31)	< 0.01	0.12 (− 0.01, 0.26)	0.074
rs439401	APOE	WHR	CRP	0.64 (0.39, 0.88)	< 0.01	0.05 (0.01, 0.10)	0.024	0.69 (0.44, 0.94)	< 0.01

Both variants rs11208712 and SNP rs439401 (APOE) illustrated direct association with CRP after adjusting for phenotypes that SNP was primarily associated in prior studies. Results suggest biologic pleiotropy with independent associations with both inflammation and obesity. SNP rs11208712 (LEPR) was not associated with BMI after adjusting for mediator (CRP), albeit missing on nominal significance by a close margin

## Discussion

We performed pleiotropy analyses on univariate meta-analyzed GWAS of BMI, WHR and CRP, as measures of overall and central adiposity and inflammation respectively, using data from self-identified Hispanics in PAGE cohort. We identified 7 independent signals with potential for pleiotropy, 2 of which were replicated with CCHC population including rs11208712 and rs439401.

The lead variant on chromosome 1 (rs11208712) is intergenic and the closest gene is leptin receptor (*LEPR*), a gene linked to obesity [57, 58]. Although this SNP has not previously been associated with inflammation, another SNP (rs4655582) which is a close proxy of this variant (*R2* > 0.9) was associated with acute-phase serum amyloid A proteins (A-SAA) [59]. This family of apolipoproteins is secreted during the acute phase of inflammation [60]. Increased expression of this proinflammation lipolytic adipokine is suggested to play a role in systemic inflammation, free fatty acid production and is proposed as a link between obesity and CVD risk factors including insulin resistance and atherosclerosis [61]. Mediation analyses did not suggest significant association with adiposity once adjusted for mediator (CRP).

The only replicated likely pleiotropic SNP, rs439401 overlapping *APOE* gene is reported in association with different lipid markers. Analyses suggested higher gene expression levels associated with this SNP in adipose, skin, liver, thyroid, and adrenal tissues. Overall, several genetic variants in *APOE* has been widely related to pro-inflammatory measure [62, 63], and obesity [64]. One recent meta-analysis also supported strong correlation between better known *APOE* variants and CVD, especially CHD [65]. Although rs439401 is less interrogated compared to other *APOE* SNPs, but we found a study linking the SNP with high blood pressure [66], and another linking SNP’s allelic variation to poor metabolic profile (obesity, insulin resistance and high triglyceride level [67]. SNP rs584007, a variant in the same region in tight LD with rs439401, is frequently associated with CRP level [68]. rs439401 is also associated with lower risk of Alzheimer’s disease [69], and appears to be functionally independent of another *APOE* SNP (rs429358) which is the most widely studied Alzheimer’s disease variant (*R2* ~ 0.1).

Mediation analyses illustrated a positive indirect association with CRP independent of WHR, and indirect positive association via WHR, and robust total positive association, consistent with prior suggestions. Interestingly, the SNP was also proposed as potentially pleiotropic in another study due to association with inflammatory reactions in leprosy through regulatory effects on lipid metabolism [70]. Authors suggested that SNPs’ risk allele is associated with low *APOE* gene expression level, leading to increased plasma lipoprotein level that facilitates survival of mycobacterium leprae in the skin and hence sustained inflammatory lesions. Increased lipoprotein is a known risk factor for arterial inflammation [71], but results from our mediation analyses suggest a concurrent and independent association with inflammation as well.

Inadequate power with replication set was a notable limitation to our approach as the remaining 5 likely pleiotropic SNPs did not replicate in CCHC. However, review of the literature suggests strong candidates for pleiotropy and larger replication sample set may signify their potentials. For example, the SNP on chromosome 12 (rs1169288) that overlaps *HNF1A* had previously been reported as a pleiotropic variant affecting cardiometabolic traits and CRP levels [72]. The FTO variant on chromosome 16 (rs11642015) is reported as pleiotropic SNP for inflammation and lipid traits [73], and associated with BMI adjusted diabetes [74].

The precise characterization of pleiotropic signals requires insight from joint studies of genetics and gene expression, particularly in the tissues of interest for the traits interrogated. However, functional annotation evaluation was able to provide suggestive evidence for possible functional roles associated with likely causal variants. For instance, mesenchymal cell H3K4me3 promoter annotation for rs439401 was suggestive of potential pleiotropic effects at the protein level. It also should be noted that annotation tools used for this study were primarily derived using European populations and may harbors limitations when used for functional annotation of variants in an admixed group like Hispanics.

Another notable limitation was our consideration of only one self-reported Mexican American study population for replication. While all PAGE study participants self-reported Hispanic/Latino ethnicity, a large range of study participants reported backgrounds from South and Central American populations. For example, study participants self-reported Dominican, Puerto Rican, Cuban, and Chilean background, among many others. It is well known that self-reported Hispanic/Latino populations display extensive ancestral diversity from Europe, Asia, Africa, and Native America [75, 76]. Thus, our lack of replication of some discovery genetic signals may have been influenced by the limited ancestral diversity CCHC study participants, when compared to the total PAGE population. However, we do have great confidence in our replicated signals, which were common in both study populations. Finally, caution should be exercised with causal interpretation of observed pleiotropic association which require longitudinal and randomized assessments.

The present study also has major strengths. This study is the first comprehensive assessment of pleiotropic associations between adiposity and inflammation traits in a self-identified Hispanics population. The results provide suggestive evidence for the regulatory effects of identified genetic variants on metabolic pathways and highlight the complexity and interrelatedness of seemingly independent phenotypic traits. The intentional selection of a genetically tri-admixed Hispanics population [76] with shorter haplotypes compared with European ancestry (EA) populations [77] greatly narrowed the number of SNPs identified for causal evaluations [78, 79], and allowed isolation of pleiotropic loci potentially specific to Hispanic/East Asian populations.

In conclusion, our study characterized potential pleiotropy between inflammation and adiposity susceptibility variants in self-identified Hispanics. We found 2 variants with evidence for biologic pleiotropy. The lead variant on *APOE* (rs439401) and rs11208712 were found to be harbor pleiotropic association with adiposity and inflammation. Particularly, both loci illustrate significant and concurrent associations with lipoproteins levels and inflammatory markers’ variations which indicate a common functional pathway with overlapping molecular underpinning. Additional loci with similar regulatory patterns were also isolated, though study lacked power to replicate associations. Results from this study demonstrate the importance of conducting multi-trait analyses, for improving our understanding of the etiology of the various metabolic pathways that regulate cardiovascular disease development.

## Supplementary Information

**Additional file 1**. Conceptual framework for adiposity and inflammation pleiotropy.

**Additional file 2**. Analytical framework for mediation analysis. Mediating phenotype is the known associated trait with SNP. Genetic Variant is assume to have multi-trait effect if the direct, mediated and total effect all exceed statistical significance level.

**Additional file 3**. Association of PAGE derived multi-trait significant SNPs with phenotypic domains.

**Additional file 4**. Tissue specific messenger RNA (mRNA) expression levels associated with PAGE derived multi-trait associated SNPs.

**Additional file 5**. Supplementary Tables.

**Additional file 6**. Appendix

## Data Availability

PAGE accession numbers are: phs000356 PAGE collectively; phs000223 PAGE-ARIC and phs000280 ARIC Cohort; phs000555 PAGE-HCHS/SOL and phs000810 HCHS/SOL Cohort; phs000220 PAGE-MEC; phs000227 PAGE-WHI and phs000200 WHI Cohort; phs000925 PAGE-IPM-BioMe. Summary of study results of meta-analyses and aSPU multi-trait analyses will be made available on dbGap repository upon publication approval.
