# Supplementary material for: Genetic pleiotropy underpinning adiposity and inflammation in self-identified Hispanic/Latino populations
Source: BMC Med Genomics. 2022 Sep 10;15:192. doi: 10.1186/s12920-022-01352-3 (PMC9464371; doi:10.1186/s12920-022-01352-3)
Supplement: Supplementary file 1 — Additional file 1. Conceptual framework for adiposity and inflammation pleiotropy. [file 12920_2022_1352_MOESM1_ESM.docx]

**Appendix**

**Section 1.**

**Fig 1.** The population substructure present in the multi-ethnic sample of PAGE shows complex patterns.

**A)**. Here we show principal components (PC) 1 and 2 to illustrate major patterns of variation, stratified by self-identified race/ethnicity. Individuals denoted by orange self-identified as ‘Other’.


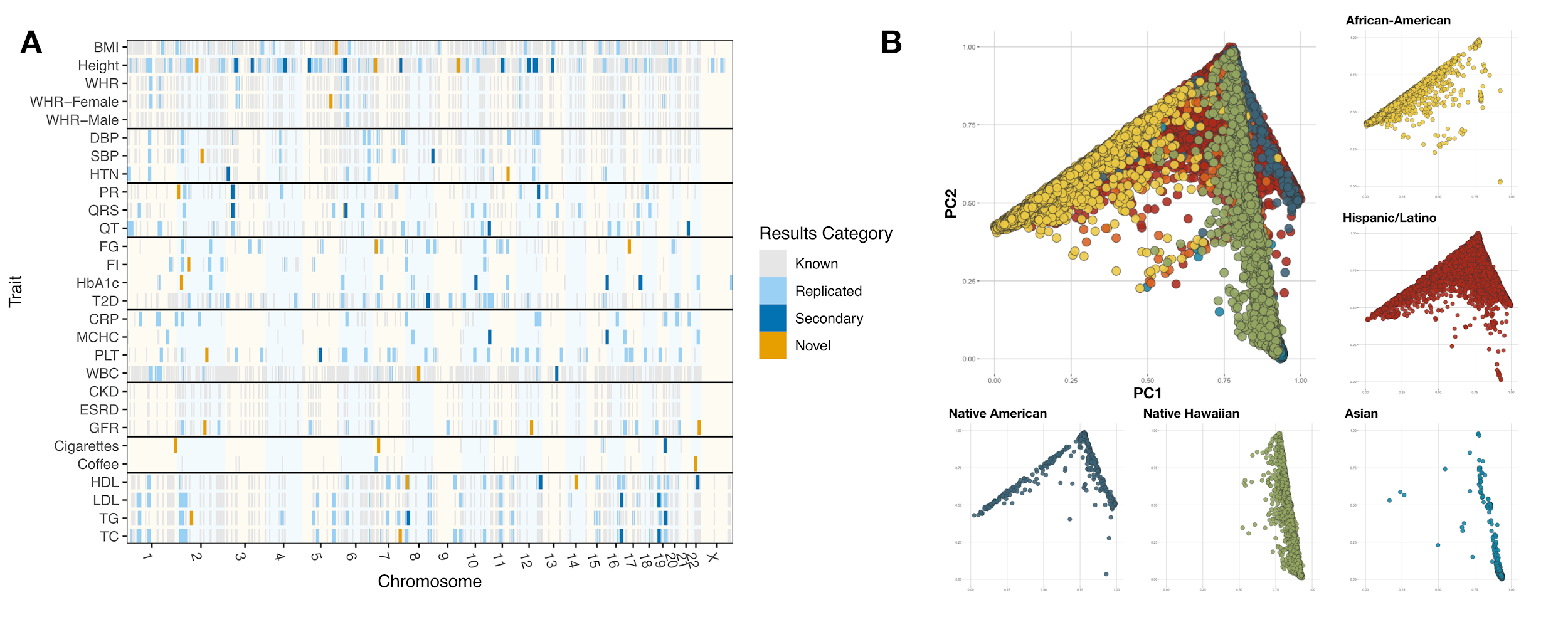


**EAST**

**ASIA**

**AMERICAS**

**EUROPE**

**AFRICA**

**B)**. Here we show principal components (PC) 1 and 2 to show major patterns of ancestry markers variation among self-identified Hispanic/Latinos in PAGE.


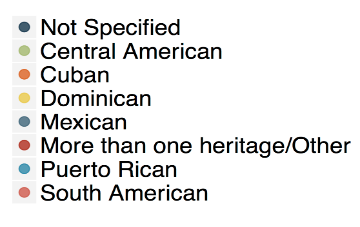

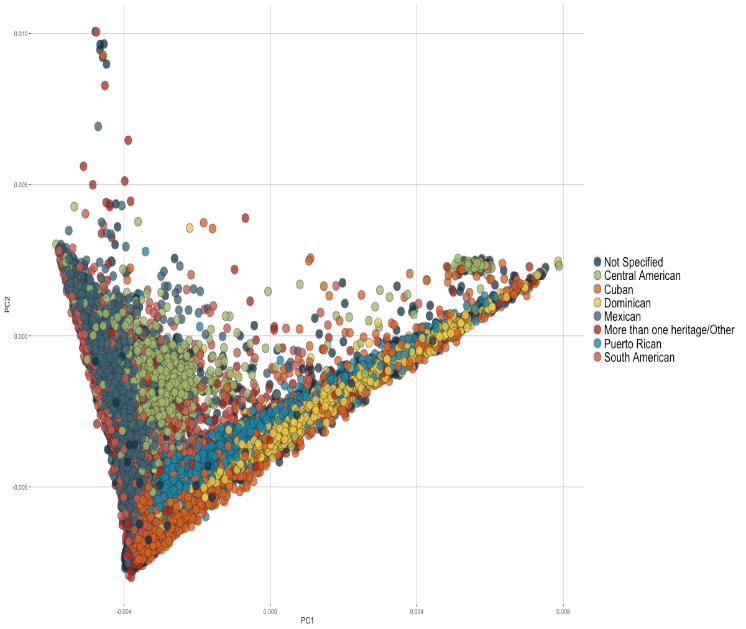


**PC2**

**PC1**

0.008 0.004 0.000 -0.004

**Fig 2.** Projection of the first three principal components (PCs) for Cameron County Hispanic Cohort (CCHC) over 1000 Genome individuals for ancestry inference. Nearly all individuals cluster with American ancestry population, which entail Mexican and Native ancestry groups. (Note: American (AMR), East Asian (EAS), African (AFR), European (EUR), Southeast Asian (SAS)).

**
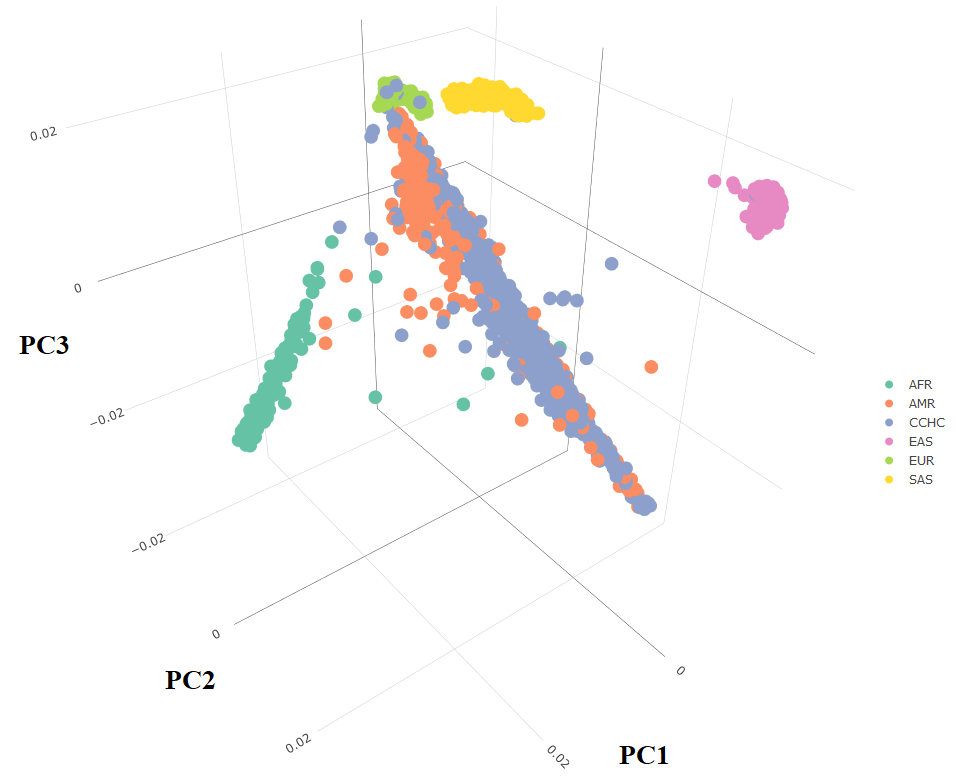
**

**Section 2.**

Effective sample size (ESS) for each SNP was calculated as:

$$ESS=\frac{imputation or genotype quality (\%)}{(heterogeneity degree of freedom+1)}\times2\times alternative allele frequency \times\left( 1-alternative allele frequency \right)\times informative number of indivudals with alleles$$
