# Supplementary figures and images for: Genetic pleiotropy underpinning adiposity and inflammation in self-identified Hispanic/Latino populations
Source: BMC Med Genomics. 2022 Sep 10;15:192. doi: 10.1186/s12920-022-01352-3 (PMC9464371; doi:10.1186/s12920-022-01352-3)

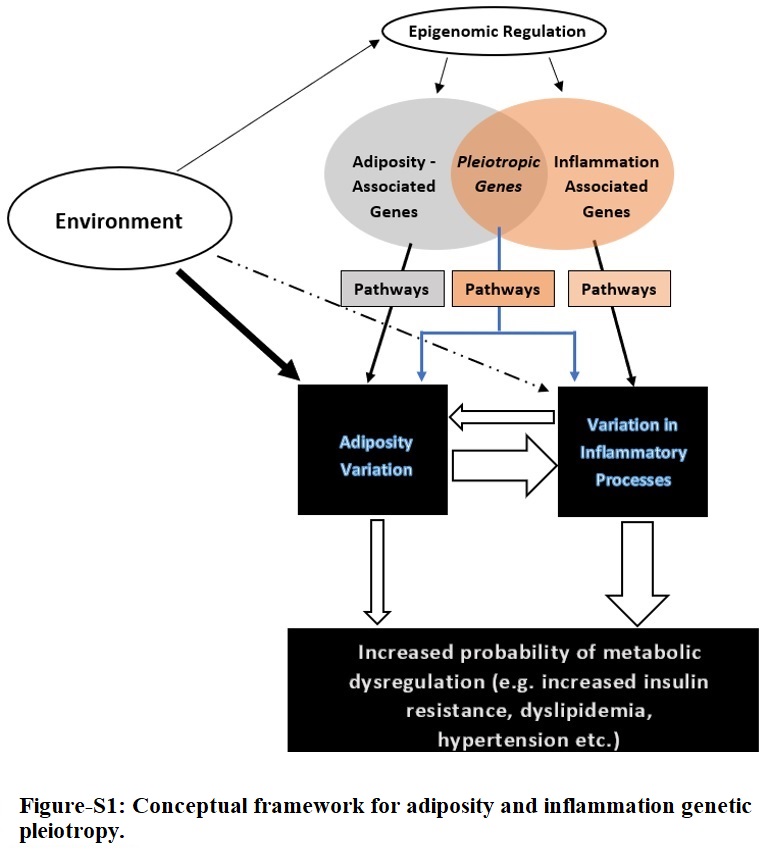

Supplement: Supplementary file 2 — Additional file 2. Analytical framework for mediation analysis. Mediating phenotype is the known associated trait with SNP. Genetic Variant is assume to have multi-trait effect if the direct, mediated and total effect all exceed statistical significance level. [file 12920_2022_1352_MOESM2_ESM.jpg]

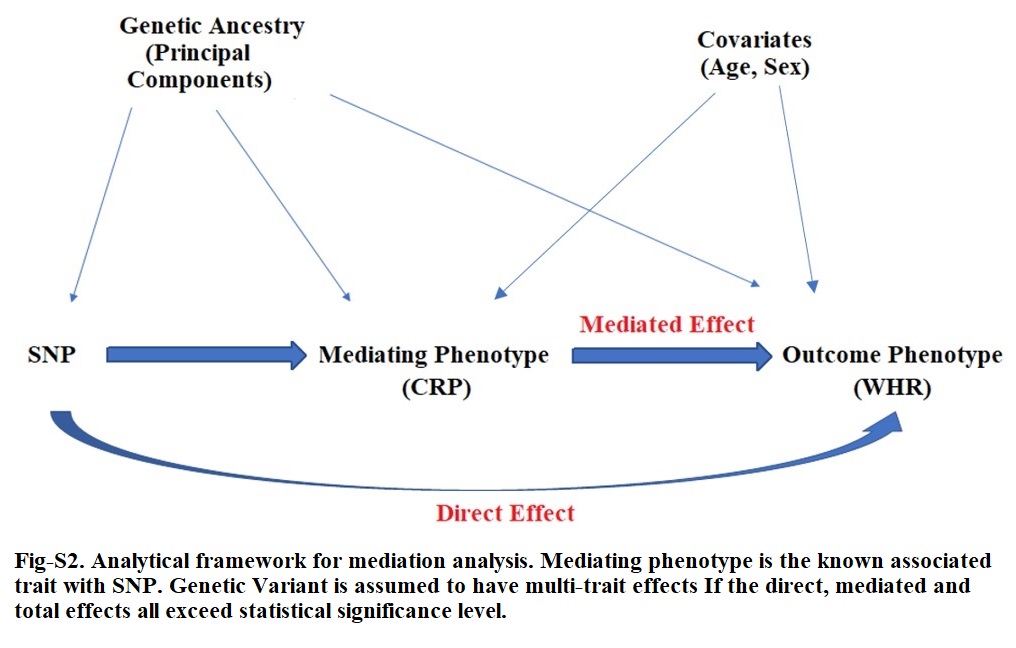

Supplement: Supplementary file 3 — Additional file 3. Association of PAGE derived multi-trait significant SNPs with phenotypic domains. [file 12920_2022_1352_MOESM3_ESM.jpg]

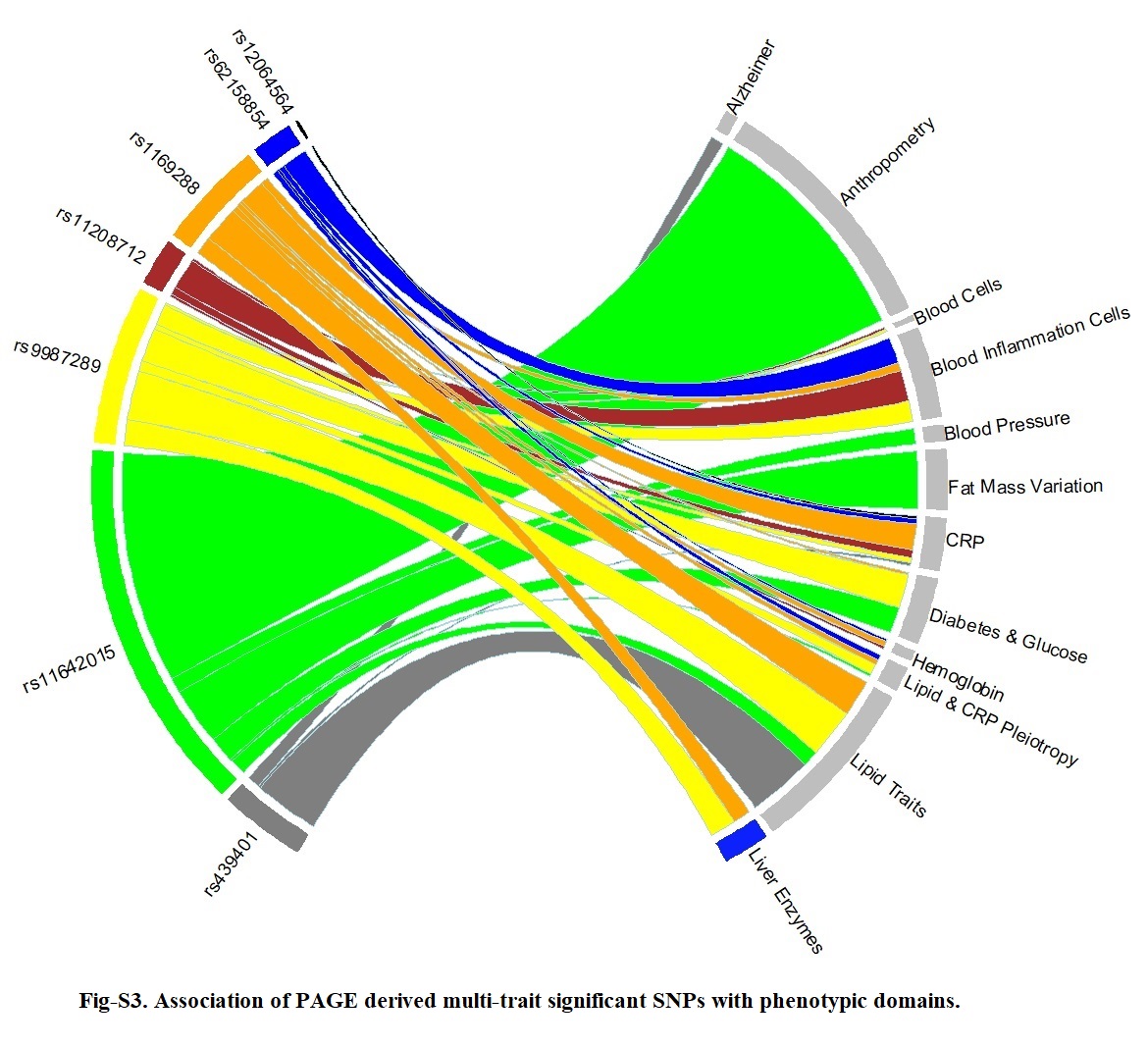

Supplement: Supplementary file 4 — Additional file 4. Tissue specific messenger RNA (mRNA) expression levels associated with PAGE derived multi-trait associated SNPs. [file 12920_2022_1352_MOESM4_ESM.jpg]

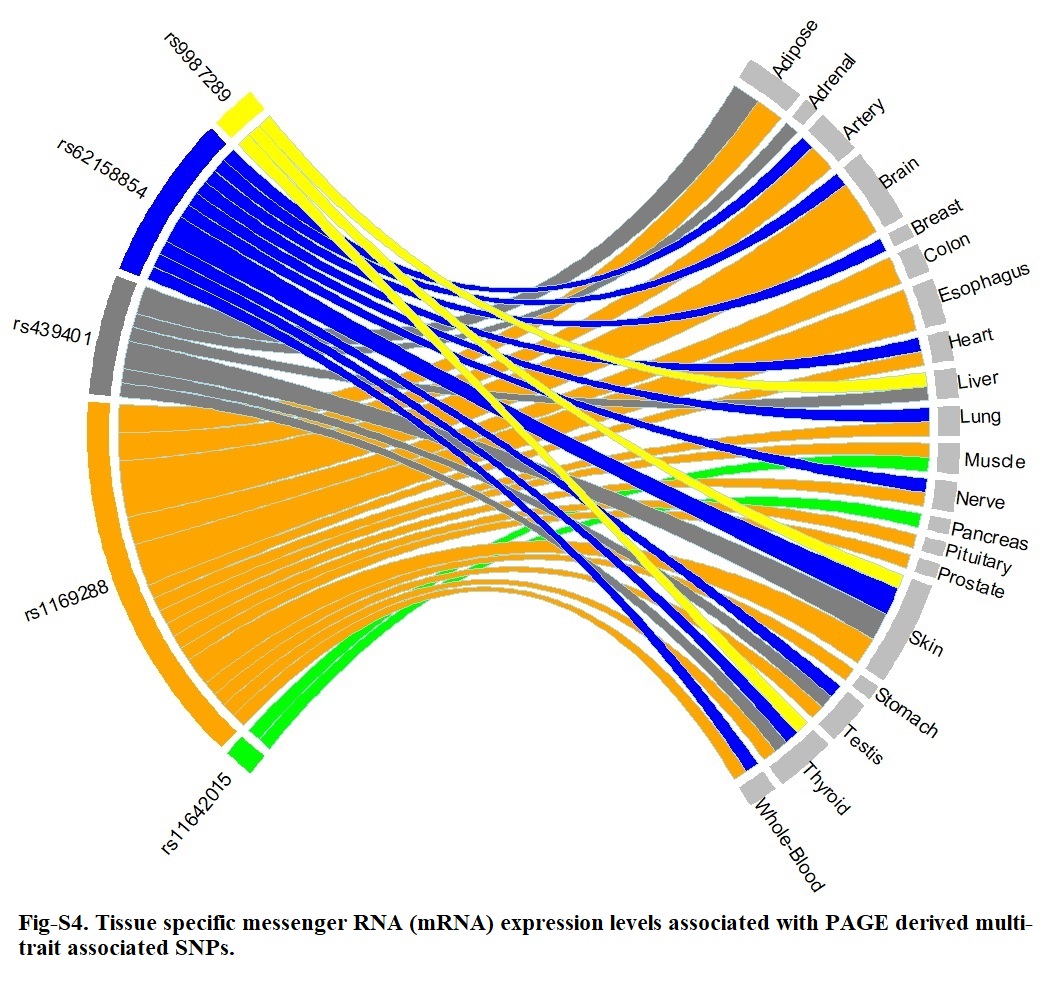

Supplement: Supplementary file 6 — Additional file 6. Appendix [file 12920_2022_1352_MOESM6_ESM.jpg]
